# SLX4 Assembles a Telomere Maintenance Toolkit by Bridging Multiple Endonucleases with Telomeres

**DOI:** 10.1016/j.celrep.2013.08.017

**Published:** 2013-09-05

**Authors:** Bingbing Wan, Jinhu Yin, Kent Horvath, Jaya Sarkar, Yong Chen, Jian Wu, Ke Wan, Jian Lu, Peili Gu, Eun Young Yu, Neal F. Lue, Sandy Chang, Yie Liu, Ming Lei

**Affiliations:** 1 State Key Laboratory of Molecular Biology, Institute of Biochemistry and Cell Biology, Shanghai Institutes for Biological Sciences, Chinese Academy of Sciences, 320 Yueyang Road, Shanghai 200031, China; 2 National Center for Protein Science Shanghai, Institute of Biochemistry and Cell Biology, Shanghai Institutes for Biological Sciences, Chinese Academy of Sciences, 320 Yueyang Road, Shanghai 200031, China; 3 Howard Hughes Medical Institute, University of Michigan Medical School, 1150 W. Medical Center Drive, Ann Arbor, MI 48109, USA; 4 Department of Biological Chemistry, University of Michigan Medical School, 1150 W. Medical Center Drive, Ann Arbor, MI 48109, USA; 5 Laboratory of Molecular Gerontology, National Institute on Aging/National Institute of Health, 251 Bayview Drive, Baltimore, MD 21044, USA; 6 Graduate School of Biomedical Sciences at New Jersey Medical School, 185 South Orange Avenue, Newark, NJ 07103, USA; 7 Department of Laboratory Medicine and Pathology, Yale University School of Medicine, 330 Cedar Street, New Haven, CT 06520, USA; 8 Department of Microbiology and Immunology, W. R. Hearst Microbiology Research Center, Weill Medical College of Cornell University, New York, NY 10065, USA

## Abstract

SLX4 interacts with several endonucleases to resolve structural barriers in DNA metabolism. SLX4 also interacts with telomeric protein TRF2 in human cells. The molecular mechanism of these interactions at telomeres remains unknown. Here, we report the crystal structure of the TRF2-*b*inding *m*otif of SLX4 (SLX4_TBM_) in complex with the TRFH domain of TRF2 (TRF2_TRFH_) and map the interactions of SLX4 with endonucleases SLX1, XPF, and MUS81. TRF2 recognizes a unique HxLxP motif on SLX4 via the peptide-binding site in its TRFH domain. Telomeric localization of SLX4 and associated nucleases depend on the SLX4-endonuclease and SLX4-TRF2 interactions and the protein levels of SLX4 and TRF2. SLX4 assembles an endonuclease toolkit that negatively regulates telomere length via SLX1-catalyzed nucleolytic resolution of telomere DNA structures. We propose that the SLX4-TRF2 complex serves as a double-layer scaffold bridging multiple endonucleases with telomeres for recombination-based telomere maintenance.

## Introduction

Telomeres are highly conserved chromosome end structures, composed of the shelterin protein complex and a double-stranded tract of tandem repeats that ends in a single-stranded 3′-overhang of the G-strand in mammals (Blackburn, 2001). The 3′ single-stranded overhang has been proposed to invade the duplex region of telomeric DNAs, base-pairing with the C-strand, forming a “t-loop” configuration (Griffith et al., 1999). Shelterin consists of several proteins including duplex telomeric DNA-binding proteins TRF1 and TRF2 that anchor shelterin along the telomere repeats (Palm and de Lange, 2008). TRF1 and TRF2 are also the principle mediators that recruit many nonshelterin telomere accessory proteins including Apollo (Palm and de Lange, 2008) to telomeres (Palm and de Lange, 2008). The telomere restriction fragment homology (TRFH) domains of TRF1 and TRF2 (Li et al., 2000) directly recognize specific but distinct motifs on their interacting proteins (Chen et al., 2008).

A critical aspect of telomere maintenance is telomere length homeostasis, involving telomerase-catalyzed extension and alternative lengthening of telomeres (ALT). In addition, TRF1 and TRF2 are negative regulators of telomere length (Ancelin et al., 2002; Smogorzewska et al., 2000; van Steensel and de Lange, 1997). Although TRF1 is believed to regulate telomerase in *cis* at individual telomeres, TRF2 may control telomere length by telomere degradation (Ancelin et al., 2002). Homologous-recombination (HR)-mediated resolution of t-loop has also been proposed to be the underlying mechanism for a negative telomere length regulation process, referred to as “telomere trimming” (Pickett et al., 2009; Wang et al., 2004), which leads to deletion of large telomeric segments that are detectable as extrachromosomal telomeric circles (TCs) (Pickett et al., 2009, 2011; Wang et al., 2004). Some cancer cells maintaining telomere length via ALT exhibit abundant TCs (Henson and Reddel, 2010) and likely utilize telomere trimming to counteract HR-mediated telomere lengthening (Cesare and Reddel, 2010). Yet, the regulatory mechanism for telomere trimming is not fully understood.

A Fanconi anemia (FA) protein, SLX4 (or BTBD12 or FANCP), is a molecular scaffold that interacts with structure-specific endonucleases XPF-ERCC1, MUS81-EME1, and SLX1 (Fekairi et al., 2009; Kim et al., 2013; Muñoz et al., 2009; Svendsen et al., 2009). SLX4 also associates with TRF2 in human cells (Svendsen et al., 2009). However, the nature of this interaction and the functional significance of SLX4 at telomeres remain unknown. Here, we report that telomeric localization of SLX4 and its associated nucleases depends on the interaction between a unique HxLxP motif of SLX4 and the TRFH domain of TRF2. We propose that SLX4 together with TRF2 forms a double-layer scaffold that assembles a telomere maintenance toolkit, containing XPF, MUS81, and SLX1, where the SLX4-SLX1 module regulates telomere length via SLX1-catalyzed resolution of telomeric DNA structures.

## Results

### Telomeric Localization of SLX4 Depends on Protein Levels of SLX4 and TRF2

**Figure 1:**
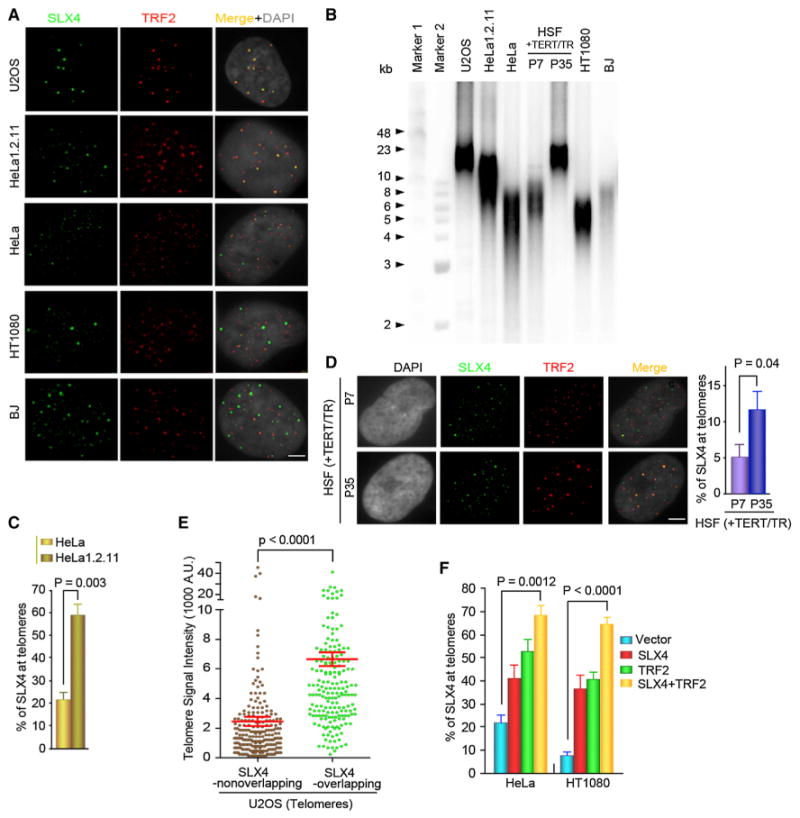
Association of SLX4 with Telomeres Is Dependent on Proteins Levels of SLX4 and/or TRF2 (A) Endogenous SLX4 and TRF2 colocalization in human cells. Bar: 5 μm. (B) Telomere length in different human cell lines by TRF analysis. (C) Percentage of SLX4 foci at telomeres in HeLa and HeLa1.2.11 cells. One hundred cells/genotype were examined. p value: two-tailed Student's test. (D) Telomeric localization of SLX4 in primary human fibroblasts induced with hTERT and hTR (long telomeres: HSF-P35; short telomeres: HSF-P7). Error bars: SD; p values: two-tailed Student's t test. (E) Signal intensity of SLX4-nonoverlapping or -overlapping telomeres. Error bars: SD. p values: two-tailed Student's t test. (F) Percentage of SLX4 foci at telomeres in HeLa and HT1080 cells transfected with SLX4 and/or TRF2. Error bars: SD; p values: two-tailed Student's t test. See also Figure S1.

To investigate the involvement of SLX4 in telomere maintenance, we examined telomeric localization of endogenous SLX4 in different human cell lines, including telomerase-negative ALT cells (U2OS, WI38[VA13-2RA], and GM847), telomerase-positive cancer cells (HT1080, HeLa, and its subclone HeLa1.2.11), and primary cells (BJ and MRC5). Indirect immunofluorescence (IF) showed that SLX4 formed discrete nuclear foci in all of these cells (Figures 1A and S1A; data not shown). Unlike the primary (BJ) and telomerase-positive (HT1080 and HeLa) cancer cells, the majority of the SLX4 foci in ALT and HeLa1.2.11 cells colocalized with TRF2 and telomeres (Figures 1A and S1A). The association of SLX4 with telomeres in U2OS and HeLa1.2.11 cells was also confirmed by chromatin immunoprecipitation (ChIP) (Figure S1B). Because telomeres in ALT cells are substantially longer than those in other cells and HeLa1.2.11 cells possess longer telomeres than its parental HeLa cells (Figure 1B), we reasoned that SLX4 may preferentially localize to longer telomeres. Indeed, SLX4 increasingly localized to telomeres in telomerase-positive cells with longer telomeres (Figures 1C and 1D; Supplemental Information). Notably, the few telomeres in the ALT (U2OS) cells that did not colocalize with SLX4 foci also exhibited substantially weaker signals than the majority of SLX4-overlapping telomeres (Figure 1E), further suggesting that SLX4 tends to be targeted to longer telomeres. The amount of SLX4 at individual telomeres was also dependent on the protein levels of SLX4 and TRF2 (Figures 1F and S1C; Supplemental Information), which in many cells positively correlate with longer telomere length.

### A Short Motif on SLX4 Mediates the SLX4-TRF2 Interaction Essential for Telomeric Localization of SLX4

**Figure 2:**
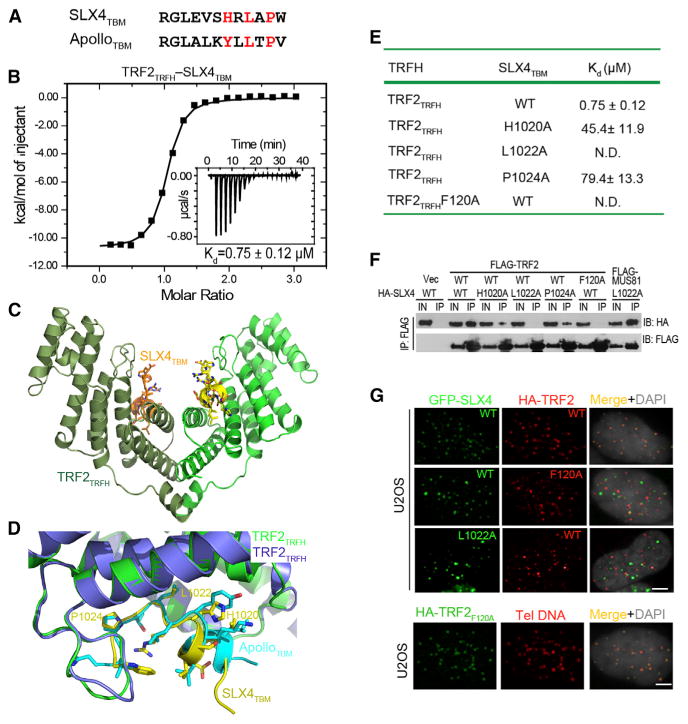
The SLX4-TRF2 Interaction Is Critical for Telomeric Localization of SLX4 (A) Sequence comparison between SLX4_TBM_ and Apollo_TBM_. Key residues that mediate the interaction with TRF2_TRFH_ are highlighted in red. (B) ITC measurement of the SLX4_TBM_-TRF2_TRFH_ interaction. Inset shows the ITC titration. (C) Overall structure of the SLX4_TBM_-TRF2_TRFH_ complex. TRF2_TRFH_ and SLX4_TBM_ are colored in green and yellow, respectively, in one monomer, and dark green and orange, respectively, in the other. (D) Superposition of the TBM peptide-binding sites in the SLX4_TBM_-TRF2_TRFH_ and Apollo_TBM_-TRF2_TRFH_ complexes. TRF2_TRFH_ is colored in purple and green in the SLX4_TBM_-TRF2_TRFH_ and Apollo_TBM_-TRF2_TRFH_ complexes, respectively. (E) Equilibrium dissociation constants (K_d_) of WT and mutant SLX4_TBM_-TRF2_TRFH_ interactions measured by ITC. N.D., not detectable. (F) Co-IP of WT and mutant SLX4 and TRF2 in 293T cells. Lanes marked “IN” contain 5% of the input lysate used for IPs. (G) Nuclear localization of WT and mutant SLX4 and TRF2. Telomeric DNA was detected by the Cy3-labeled (CCCTAA)_3_ PNA probe. Bar: 5 mm. See also Figure S2.

TRF2 interacts with the middle region of SLX4 (Svendsen et al., 2009). We found that SLX4 binds to the TRFH domain of TRF2 (TRF2_TRFH_) by yeast two-hybrid (Y2H) assay (data not shown). Various fragments of SLX4 were evaluated for their ability to interact with TRF2_TRFH_ by Y2H and isothermal titration calorimetry (ITC) assays (Figures S2A–S2E). A short fragment of SLX4 consisting of residues 1014–1028 (SLX4_1014–1028_) was sufficient for binding with TRF2_TRFH_ with a K_D_ of 750 nM (Figures 2A and 2B), whereas binding enthalpy between SLX4_1014–1028_ and TRF1_TRFH_ was not measurable by ITC (Figure S2F), indicating that SLX4_1014–1028_ binding is specific for TRF2. We hereafter refer to SLX4_1014–1028_ as SLX4_TBM_ (*T*RF2-*b*inding *m*otif) (Figure 2A).

We have demonstrated that TRF2_TRFH_ specifically recognizes a short peptide motif with a signature sequence YxLxP (x: any amino acid) on its interacting proteins including Apollo (Chen et al., 2008). SLX4_TBM_ contains a modified sequence HxLxP (Figure 2A) that highly resembles the TRF2_TRFH_ binding motif of Apollo (Apollo_TBM_) (Figure 2A) (Chen et al., 2008), suggesting that SLX4_TBM_ might bind to TRF2_TRFH_ in the same fashion as Apollo_TBM_. We solved the crystal structure of the TRF2_TRFH_-SLX4_TBM_ complex at 2.05 Å resolution (Figure S2G; Table S1). The structure exhibits a 2:2 stoichiometry between TRF2_TRFH_ and SLX4_TBM_. Each SLX4_TBM_ peptide adopts an extended conformation with a short one-turn helix at the N terminus and fits into the narrow groove formed by TRF2_TRFH_ (Figure 2C). Except for the very N terminus, SLX4_TBM_ and Apollo_TBM_ are identical in overall conformation (Figure 2D). Two key hydrophobic residues in SLX4_TBM_, L1022, and P1024 bind to TRF2_TRFH_ in the same fashion as do their counterparts in Apollo_TBM_. The imidazole ring of SLX4 H1020 occupies the same position as the side chain of Apollo Y504 in the Apollo_TBM_-TRF2_TRFH_ complex (Figure 2D) (Chen et al., 2008).

To investigate the importance of the interface residues of SLX4_TBM_ or TRF2_TRFH_ in the SLX4-TRF2 interaction, we focused on four hydrophobic residues: F120 of TRF2 and H1020, L1022, and P1024 of SLX4, whose equivalents in other TRF2_TRFH_-binding proteins are critical for their respective interactions (Chen et al., 2008). ITC data showed that alanine substitutions of these residues either completely abolished (SLX4 L1022A and TRF2 F120A) or substantially impaired (SLX4 H1020A and P1024A) the SLX4-TRF2 interaction (Figures 2E, S2H, and S2I). Consistent with the ITC analysis, coimmunoprecipitation (co-IP) revealed that while SLX4_L1022A_ and TRF2_F120A_ completely disrupted the association between SLX4 and TRF2, mutations of SLX4 H1020 and P1024 to alanine retained weak interactions with TRF2 in human embryonic kidney 293T cells (Figure 2F). Furthermore, SLX4_L1022A_ was efficiently coimmunoprecipitated with MUS81 (Figure 2F) that is known to bind to the C-terminal region of SLX4 (Fekairi et al., 2009; Kim et al., 2013; Svendsen et al., 2009). Hence, the observed SLX4-TRF2 interface is specific and necessary for both in vitro and in vivo binding of SLX4 to TRF2.

We tested the relevance of the SLX4-TRF2 interaction to the telomeric localization of SLX4 in U2OS and HeLa1.2.11 cells transiently expressing wild-type (WT) or mutant GFP-SLX4 fusion protein and HA-tagged WT or mutant TRF2. IF analysis revealed a nuclear punctate staining pattern for WT SLX4 that almost completely colocalized with TRF2 (Figures 2G and S2J–S2L). In contrast, the SLX4_L120A_ and TRF2_F120A_ mutants disrupted the colocalization of SLX4 and TRF2 (Figures 2G and S2J–S2L). As both WT TRF2 and TRF2_F120A_ mutant can efficiently localize to telomeres (Figures 2G and S2J–S2L), these results demonstrate that SLX4 binding to TRF2_TRFH_ is required for telomeric localization of SLX4.

### Nuclear Foci Formation and Telomeric Localization of XPF, MUS81, and SLX1 Are Mediated by SLX4

**Figure 3:**
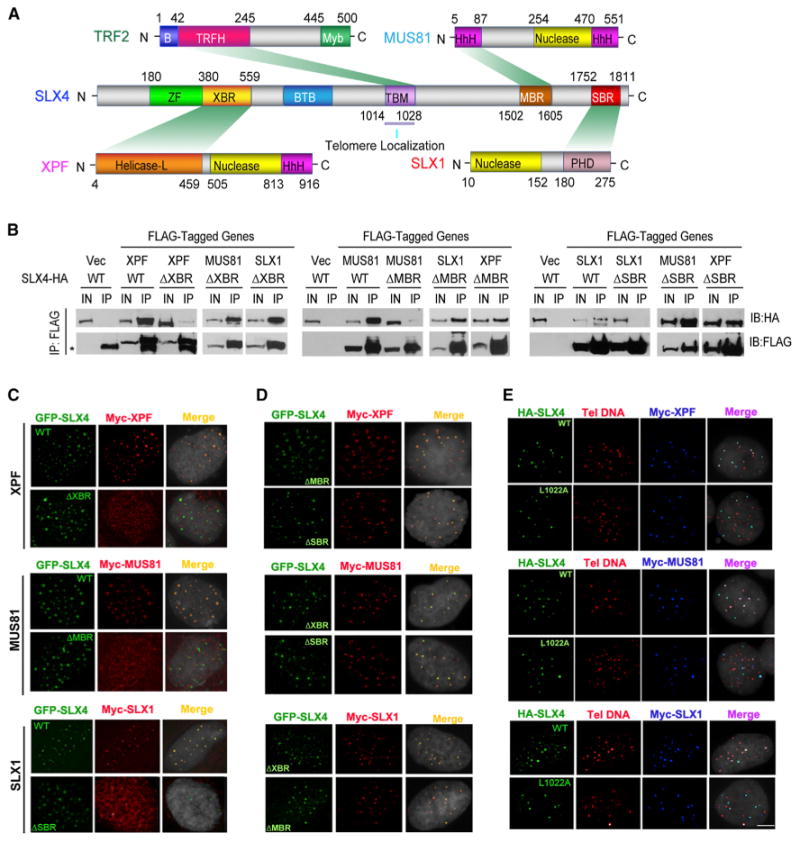
SLX4 Serves as a Scaffold to Assemble a Nuclease Toolkit at Telomeres (A) Domain organization of SLX4 and its interacting proteins. The shaded areas between SLX4 and its interacting proteins indicate the regions of the respective protein-protein interactions. B, basic domain. (B) Co-IP confirmation of SLX4-nuclease interacting regions. Lanes marked “IN” contain 5% of the input lysate used for IPs. Asterisk: nonspecific band. (C and D) IF analysis of foci formation and colocalization of the nucleases in SLX4-depleted U2OS cells expressing WT or deletion mutants of SLX4 that either disrupt (C) or preserve (D) the respective interactions. (E) IF-telomere FISH analysis of foci formation and colocalization of the nucleases with telomeric DNA in SLX4-depleted U2OS cells expressing WTSLX4 or TRF2-binding-deficient mutant SLX4_L1022A_. See also Figure S3.

SLX4 contains modules that interact with other protein factors, including XPF, MUS81, and SLX1 (Fekairi et al., 2009; Kim et al., 2013; Muñoz et al., 2009; Svendsen et al., 2009). Using Y2H assay, we mapped three nonoverlapping fragments of SLX4 that interact with XPF, MUS81, and SLX1 (Figures 3A and S3A–S3C), hereafter referred to as XBR (*X*PF-*b*inding *r*egion), MBR (*M*US81-*b*inding *r*egion), and SBR (*S*LX1-*b*inding *r*egion), respectively (Figure 3A). Co-IP showed that the deletion mutants of SLX4 (SLX4_ΔXBR_, SLX4_ΔMBR_, and SLX4_ΔSBR_), each lacking only the specific interacting region for one endonuclease, specifically abolished only the targeted interaction in cells (Figure 3B). Furthermore, IF analysis demonstrated that nuclear foci formation of XPF, MUS81, and SLX1 relied on their specific interactions with SLX4 (Figures 3C and S3D–S3F; Supplemental Information).

Because TRF2 bridges SLX4 with telomeres, we tested whether the SLX4-TRF2 interaction is critical for telomeric localization of the SLX4-interacting nucleases. We utilized either the SLX4_L1022A_ or the TRF2_F120a_ mutant, each of which can independently abolish the SLX4-TRF2 interaction (Figures 2E–2G and S2H–S2L), and individually expressed each endonuclease with SLX4_L1022a_ (Figures 3E and S3G) or TRF2_F120a_ (data not shown) and assayed their nuclear localization. Although XPF, MUS81, and SLX1 formed discrete foci that colocalized with SLX4_L1022a_, they failed to be targeted to telomeres (Figures 3E and S3G). Also, all three nucleases failed to colocalize with TRF2_F120a_ (data not shown). Hence, SLX4 functions as a scaffold to target XPF, MUS81, and SLX1 to telomeres via its interaction with TRF2.

### SLX4 Mediates Its Affiliated Nucleases in Regulating Telomere Recombination

MUS81 has been shown to regulate T-SCE in human ALT cells (Zeng et al., 2009). Because the telomeric localization of MUS81 depends on SLX4 (Figure 3C), we asked whether SLX4 is required for telomere recombination. We depleted SLX4 in U2OS cells using small hairpin RNA (shRNA) (Figures S4A– S4C) and analyzed T-SCE by chromosome orientation (CO) fluorescence in situ hybridization (FISH) (Figures S4D and S4E). Whereas U2OS cells exhibited ∼0.2 T-SCEs/chromosome, depletion of SLX4 decreased the frequency to ∼0.04 T-SCEs/chromosome (Figure S4F), underscoring the importance of SLX4 in T-SCE. Expression of WT SLX4 (Figure S4C) elevated the level of T-SCEs to ∼0.45 T-SCEs/chromosome, thereby rescuing the defect in T-SCE caused by SLX4 depletion (Figure S4G). In contrast, expression of the TRF2-binding-deficient SLX4_L1022a_ (Figure S4C) failed to fully promote T-SCE (Figure S4G), suggesting that telomeric localization of SLX4 is crucial for T-SCE.

Because XPF, MUS81, and SLX1 are recruited to telomeres by SLX4, we examined the role of each of these endonucleases in T-SCE by expressing SLX4_ΔXbr_, SLX4_Δmbr_, or SLX4_ΔSbr_ deletion mutants in SLX4-depleted U2OS cells (Figure S4C). None of these mutants was able to rescue the T-SCE defects due to SLX4 depletion (Figure S4G), indicating that all three endonucleases are required for T-SCE in U2OS cells. Similar results were also observed in HeLa1.2.11 cells (Figure S4H). Thus, SLX4 regulates telomere recombination, which is dependent on the SLX4-TRF2 scaffold and also on the nucleases XPF, MUS81, and SLX1.

### SLX4 Negatively Regulates Telomere Length via Association with SLX1

**Figure 4:**
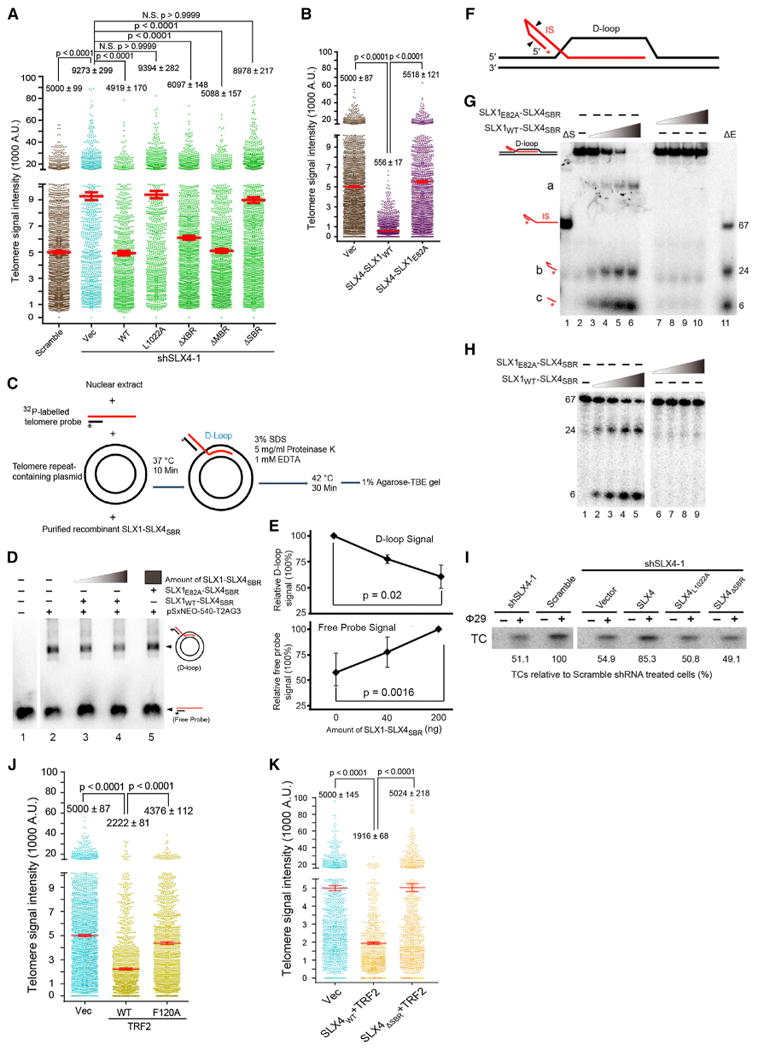
The SLX4-SLX1 Nuclease Complex Regulates Telomere Length and T-Loop Resolution (A and B) Telomere length in SLX4-depleted U2OS cells expressing WT or mutant SLX4 (A) or WT SLX4 fused to WT or nuclease dead (E82A) mutant of SLX1 (B). Mean telomere length was derived from three independent experiments. NS, nonsignificance. p values: one-way ANOVA. (C) Plasmid-based D-loop assay in the presence of purified SLX1-SLX4_SBR_ complex. (D and E) Agarose gel (D) and quantification (E) showing SLX1_WT_-SLX4_SBR_ complex-dependent change in radioactive signals corresponding to the D-loop and free probe. For each reaction, the D-loop and free probe signal were normalized to the reactions containing no (0 ng) and maximum enzyme (200 ng), respectively. Lane 1 in (D) denotes the control without telomere repeat-containing plasmid. Error bars denote variations from three independent experiments. (F) The oligonucleotide-based telomeric D-loop substrate. The invading strand (IS) was labeled with [^32^P]-γ-ATP at the 5′ end (in red). Arrows: possible cleavage sites by the SLX1-SLX4_SBR_ nuclease complex. (G and H) The telomeric D-loop substrate was incubated with increasing amounts of the SLX1_WT_-SLX4_SBR_ or SLX1_E82A_-SLX4_SBR_ complexes. The IS and cleavage products are indicated on native (G) or denaturing (H) gel. Symbols DS (lane 1): heat denatured substrate alone. ΔE (lane 11): substrate incubated with maximum amount of the WT enzyme complex, followed by heat denaturation. (I) TCA assay in SLX4-depleted U2OS cells, transiently expressing vector, WT, or mutant SLX4. The TC signal was normalized to the scramble control (lane 2) that was set for 100%. (J and K) The SLX4-SLX1 complex is a player in TRF2-mediated telomere shortening. Q-FISH analysis of telomere length in U2OS cells expressing WT or mutant TRF2 alone (J) or together with SLX4_WT_ or SLX4_DSBR_ (K). Mean telomere length was derived from three independent experiments. Error Bars: SD. NS, nonsignificance. p values: one-way ANOVA. See also Figure S5.

In mammalian cells, telomere length is maintained at or near an equilibrium point. Because SLX4 is preferentially detected at longer telomeres and interacts with nucleases in human cells (Figures 1 and 3), we investigated the role of SLX4 and its associated nucleases in telomere length regulation. U2OS cells stably expressing SLX4 shRNA were transiently transfected with either empty GFP vector or GFP-fused WT or mutant SLX4 and subsequently analyzed for telomere length using quantitative FISH (Q-FISH) analysis. SLX4-depleted cells (vec) exhibited a 2-fold increase in telomere length in comparison to the control cells (Scramble) (Figures 4A, S5A, and S5B), indicating that SLX4 is a negative regulator of telomere length. Telomere length was quickly restored to the levels of control cells, 24–48 hr after expression of WT SLX4 (Figure 4A). This suggests that the telomere lengthening in SLX4-depleted cells is attributable to SLX4 deficiency. Expression of the TRF2-binding-deficient mutant SLX4_L1022A_ could not restore telomere length in SLX4-depleted cells to that of control cells (Figure 4A), underscoring the importance of TRF2-mediated telomeric localization of SLX4 in telomere length regulation. Interestingly SLX4_ΔSBR_ failed to rescue the telomere length defect due to SLX4 depletion, whereas SLX4_ΔXBR_ or SLX4_ΔMBR_-expressing cells exhibited normal or a slightly longer average length than those in control cells (Scramble) (Figure 4A). These results support that, among the three endonucleases, SLX1 is essential for telomere length regulation in human cells.

To examine whether SLX1 regulates telomere length by nucleolytic cleavage of telomere DNAs, we utilized the nuclease-deficient E82A mutant of SLX1 (SLX1_E82A_). Because SLX1 is neither stable nor active without SLX4 (Svendsen et al., 2009), we generated chimeric constructs expressing WT or E82A SLX1 fused to SLX4, referred to as SLX4-SLX1_WT_ or SLX4-SLX1_E82A_. Expression of SLX4-SLX1_WT_ in SLX4-depleted U2OS cells caused a marked reduction in telomere length as compared to the control cells (vec). In sharp contrast, expression of SLX4-SLX1_E82A_ had no effect on telomere length (Figure 4B). Similar results were observed in HeLa1.2.11 cells (Figure S5C). These data suggest that the nuclease activity of the SLX4-SLX1 complex plays an important role in telomere length maintenance in human cells.

### The SLX4-SLX1 Complex Provides Nuclease Activity for T-Loop Resolution

Negative regulation of telomere length in human cells might involve a highly controlled process called “telomere trimming” via nucleolytic resolution of t-loops, leading to formation of TCs (Pickett et al., 2009, 2011; Wang et al., 2004). The SLX4-SLX1 complex displays robust resolution activity towards HJ substrates in vitro (Fekairi et al., 2009; Muñoz et al., 2009; Svendsen et al., 2009) and might mediate negative regulation of telomere length by catalyzing nucleolytic resolution of the t-loop. We first employed a plasmid-based D-loop assay (Verdun and Karlseder, 2006) to examine whether SLX4-SLX1 is capable of resolving D-loop, an intrinsic part of a t-loop, in vitro. In this assay, we relied on the requisite protein factors in nuclear extract to form a D-loop in the presence of a telomeric-repeat-containing plasmid (pSxNeo540-T_2_AG_3_) and a radiolabeled telomeric probe, with or without purified recombinant full-length SLX1 in complex with the C-terminal SBR domain of SLX4 (SLX1-SLX4_SBR_) (Figure 4C). With increasing amount of the SLX1_WT_-SLX4_SBR_ complex, the radioactive signal corresponding to the D-loop significantly decreased in intensity, with concomitant increase in free probe intensity, suggesting that the SLX1-SLX4 complex antagonizes or impedes D-loop formation in vitro (Figures 4D and 4E). In contrast, D-loop or free probe signal was unaltered when the catalytically deficient mutant SLX1_E82A_-SLX4_SBR_ complex was added in the assay, even at the maximum concentration (Figures 4D and 4E). Thus, the endonuclease activity of SLX1 is likely instrumental in thwarting D-loop formation.

To gain better mechanistic insight into D-loop resolution by the SLX1-SLX4 complex, we generated a model immobile telomeric D-loop structure in vitro, assembled from oligonucleotides (Figure 4F) (Opresko et al., 2004). A 67-mer invading strand (IS) that mimics the 3′-telomeric overhang was radiolabeled at the 5′ end and hybridized with the melted region of a duplex DNA to form a telomeric D-loop structure. Increasing amounts of the SLX1_WT_-SLX4_SBR_ complex resulted in disappearance of the D-loop substrate and the appearance of three bands (denoted by a, b, c), migrating below the D-loop on a native gel (Figure 4G, lanes 3–6, and Figure S5D). Bands “b” and “c” correspond to the digested and released products of the IS (Figure 4G, compare lanes 3–6 with 11), whereas band “a” very likely represents a partially resolved D-loop that remains associated with the intact IS, formation of which requires nucleolytic cleavage by SLX1, as implied from the absence of this band in the nuclease dead mutant (Figure 4G, compare lanes 3–6 with 7–10). The two cleavage products (b and c) were also visualized by a denaturing gel and are approximately 24-mer and 6-mer in length, respectively (Figure 4H, lanes 2–5). Significantly, in vivo, a nucleolytic cut on the invading 3′ -telomeric overhang would predictably result in resolution of the t-loop structure. In contrast, the D-loop substrate was completely resistant to the catalytically deficient SLX1_E82A_-SLX4_SBR_ mutant complex (Figure 4G, lanes 7–10, Figure 4H, lanes 6–9, and Figure S5D). These data support that the endonuclease activity of SLX1 is necessary for the cleavage and release of the IS and hence for proper resolution of the D-loop structure in vitro.

We reasoned that, if the SLX1-SLX4 complex nucleolytically resolves D-loops in vitro, this might translate into enhanced TC formation in vivo. We analyzed TC formation in U2OS cells depleted of SLX4 by employing a F29 DNA-polymerase-dependent T-circle amplification (TCA) assay (Figures 4I and S5E) (Zellinger et al., 2007). A substantial decrease in TC formation was observed in SLX4-depleted cells (Figure 4I, shSLX4-1), as compared to control shRNA-treated cells (scramble), suggestive of the importance of SLX4 in TC biogenesis in human cells. TC formation was nearly completely rescued by WT SLX4 but not the SLX1-binding-deficient mutant SLX4_ΔSBR_ (Figure 4I), further supporting that SLX1 plays an important role in the generation of TCs. We also observed that expression of the TRF2-binding-deficient mutant SLX4_L1022A_ in SLX4-depleted U2OS cells failed to rescue TC-formation to control levels, underscoring the importance of SLX4-TRF2 interaction in SLX1-dependent TC formation and is consistent with the colocalization data that targeting SLX1 to telomeres is dependent on the SLX4 and TRF2 interaction (Figure 3E).

### TRF2-Mediated Telomere Shortening Relies upon the SLX4-SLX1 Complex

Expression of TRF2 causes rapid loss of telomeres in ALT cells (Figure 4J) (Ancelin et al., 2002). Of note, overexpression of the TRF2_F120A_ mutant did not alter telomere length (Figure 4J), suggesting that factors that bind to the TRFH peptide-binding site of TRF2 contribute to telomere shortening. Because SLX4 interacts with TRF2 via the TRFH peptide-binding site and depletion of SLX4 leads to telomere lengthening (Figure 4A), SLX4 might be required for TRF2-mediated telomere shortening in ALT cells. U2OS cells were cotransfected with mCherry-fused WT TRF2 and GFP-fused WT SLX4 or the SLX1-binding-deficient mutant SLX4_ΔSBR_. Cells positive for GFP and mCherry signals were analyzed for telomere length by Q-FISH. Coexpression of TRF2 and SLX4 led to rapid telomere loss (Figures 4K). In contrast, coexpressing TRF2 and SLX4_ΔSBR_ had no detectable effect on telomere length (Figures 4K and S5F). A similar result was also observed in HeLa1.2.11 cells (Figure S5G). Thus, TRF2-mediated rapid telomere shortening requires SLX1 that is recruited to telomeres via SLX4.

For additional details, see the Extended Results.

## Discussion

In this report, we provide structural, molecular, and cellular evidence that SLX4 through its interaction with TRF2 functions as a “double-layer scaffold” to organize a multinuclease “telomere maintenance” toolkit. Assembly of this toolkit requires the SLX4-TRF2 interaction and three nonoverlapping motifs of SLX4 (XBR, MBR, and SBR) to recruit endonucleases XPF, MUS81, and SLX1, respectively, to the SLX4 foci. An advantage of this “double-layer” scaffold is that it can more efficiently recruit multiple proteins simultaneously to telomeres with fixed stoichiometry than a “single-layer” architecture in which all factors would be recruited by interactions with TRF2 alone. Another advantage is that SLX4 may function not only as the binding platform for the endonucleases, but also as a coordinator, orchestrating complicated processes at telomeres in a regulated manner. Given that all three endonucleases are required for proper T-SCE and that each nuclease exhibits different in vitro substrate preference (Fekairi et al., 2009; Muñoz et al., 2009; Svendsen et al., 2009), it is likely that T-SCE requires activities of all three nucleases, coordinated by SLX4. In fact, it has been proposed that resolution of HJs in vivo by the SLX4-nuclease complex may require SLX1 to first create a nicked intermediate that is then acted upon by MUS81 (Svendsen et al., 2009).

TRF1 and yeast telomeric protein Rap1 function as *cis*-acting negative regulators of telomere length (Bianchi and Shore, 2008; van Steensel and de Lange, 1997). TRF2 is also a negative regulator of telomere length and its expression causes rapid loss of telomeres in human ALT cells (Ancelin et al., 2002). Here, we unveil the SLX4-SLX1 nuclease complex assembled on telomeres as a player in TRF2-mediated negative telomere length regulation in *cis* and propose the following model (Figure S5H). TRF2 binds to telomeric DNA and functions as a measuring device to assess telomere length. Longer telomeres are bound by larger amount of TRF2, which subsequently recruits more SLX4 to telomeres. The double-layered SLX4-TRF2 platform then assembles a nuclease toolkit at telomeres for HR-mediated telomere recombination, including T-SCE and t-loop resolution. Nuclease SLX1 catalyzes nucleolytic cleavage of telomere DNA and leads to “telomere trimming.”We speculate that the telomere trimming process would continue until telomeres no longer bind sufficient TRF2 and hence the SLX4-SLX1 complex. We propose that SLX4-TRF2-assembled nuclease toolkit helps maintain an average equilibrium telomere length thus preventing telomere overlengthening. Our study reveals a major difference between TRF1 and TRF2 *cis*-acting mechanisms of telomere length regulation. Although TRF1 regulates telomere length via inhibiting telomerase activity, TRF2 recruits endonucleases via SLX4 to regulate telomere length. Because SLX4-TRF2-dependent telomere shortening occurs in both HeLa1.2.11 and ALT cells, nucleolytic cleavage of telomere DNAs by the SLX4-nuclease complex may be one of the common telomere length homeostasis mechanisms employed at long telomeres.

## Experimental Procedures

### Protein Expression, Purification, Crystallization, and Structure Determination

Proteins were expressed in *E. coli* BL21(DE3) or B834(DE3). Human TRF2_TRFH_ (residues 42–245), TRF1_TRFH_ (residues 65–267), and SLX4_TBM_ (residues 1014– 1028), were purified by Ni-NTA (QIAGEN) metal affinity, followed by gel-filtration chromatography. The SLX1_WT or E82A-SLX4SBR_ complexes were purified by tandem affinity steps (Ni-NTA and glutathione Sepharose), followed by gel filtration. Crystals of the SLX4_TBM_-TRF2_TRFH_ complex were grown by sitting-drop vapor diffusion at 4°C. Structure of the TRF2_TRFH_-SLX4_TBM_ complex was solved by molecular replacement.

### Telomere Detection Assays

Q-FISH (Zijlmans et al., 1997) or CO-FISH (Bailey et al., 2004) were used to measure telomere length and T-SCE, respectively. For IF-telomere FISH, cells were stained with primary and subsequently Alexa-Fluor-labeled secondary antibodies, followed by fixation and telomere-FISH (Wang et al., 2010). TCA (Zellinger et al., 2007) was used to detect telomere circles. Plasmid (Verdun and Karlseder, 2006) or oligonucleotide-based (Opresko et al., 2004) assays were used to detect D-loop resolution.

For additional details, see the Extended Experimental Procedures.

## Supplementary Material

01 **Figure S1. SLX4 Foci Colocalize with Telomeres, Related to** Figure 1 (A) SLX4 forms discrete foci that colocalize with telomeric DNA and TRF2 in ALT cells. IF and IF-telomere FISH were performed using anti-SLX4 and anti-TRF2 antibodies and the PNA (CCCTAA)_3_ probe. (B) ChIP analysis of SLX4 at telomeric DNA or a control locus Alu in U2OS and HeLa1.2.11 cells. (C) Western blot analysis of endogenous SLX4 expression in human cells.
**Figure S2. Characterization of the SLX4-TRF2 Interaction, Related to** Figure 2 (A) SLX4 constructs used for mapping the interaction with TRF2. AD: activation domain. BD: DNA-binding domain. (B–D) Yeast two-hybrid assay. SLX4 directly interacts with TRF2 but not RAP1 (B); Identification of the specific interaction regions of SLX4 and TRF2 (n = 3; error bars, standard deviations) (C-D). (E and F) ITC measurement of protein-protein interactions. TRF2_TRFH_ and various SLX4 fragments (E); TRF1_TRFH_ and SLX4_TBM_ peptide (St8) (F). K_d_: equilibrium dissociation constant; N.D.: not detectable by ITC. (G) Simulated annealing omit map of the SLX4_TBM_ peptide in the SLX4_TBM_-TRF2_TRFH_ complex. The omit map is contoured at 2.5 s and colored in cyan. The SLX4_TBM_ peptide is shown in magenta stick model and TRF2_TRFH_ in ribbon model. (H and I) ITC measurement of mutant SLX4_TBM_-TRF2_TRFH_ interactions. SLX4_TBM_ mutants and wild-type TRF2_TRFH_ (H); wild-type SLX4_TBM_ and TRF2_TRFH_ F120A mutant (I). K_d_: equilibrium dissociation constant; N.D.: not detectable by ITC. (J) SLX4_L1022A_ or TRF2_F120A_ do not co-localize (IF), even though TRF2_F120A_ mutant localizes to telomeric DNA (IF-telomere FISH). HeLa1.2.11 cells transiently expressing HA-tagged TRF2_F120A_ were co-transfected with either GFP-WT or mutant SLX4 fusion proteins. IF and IF-telomere FISH were performed using anti-HA antibody and the PNA (CCCTAA)_3_ probe. (K) Endogenous SLX4 colocalizes with wild-type TRF2, but not TRF2_F120A_mutant. U2OS and HeLa1.2.11 cells were transiently transfected with either HA-tagged wild-type or the F120A mutant of TRF2. IF was conducted with anti-SLX4 and anti-HA antibodies. (L) Quantification of SLX4 foci overlapping with telomeres and vice versa in U2OS cells transfected with WT or mutant SLX4 and TRF2. A total of 100 cells per each genotype were examined (related to Figure 2G). Error bars represent standard deviations. p values: two-tailed Student's test.
**Figure S3. SLX4 and Its Interaction with TRF2 Are Required for Foci Formation and Telomeric Localization of Endonucleases XPF, MUS81, and SLX1, Related to** Figure 3 (A–C) Yeast two-hybrid assay was used to characterize the interactions of SLX4 with XPF (A), MUS81 (B), and SLX1 (C). (D) Endogenous XPF, MUS81, and SLX1 form discrete foci that colocalize with endogenous SLX4 foci and telomeric DNA in U2OS cells. IF-telomere FISH was performed using anti-SLX4, anti-XPF, or anti-SLX1 antibodies and the PNA (CCCTAA)_3_ probe. (E and F) Foci formation of the endonucleases requires their respective interactions with SLX4 (E), each of which is specific (F). IF was performed in HeLa1.2.11 cells transiently expressing GFP-SLX4 wild-type or deletion mutants together with Myc-tagged XPF, MUS81, or SLX1. (G) The L1022A mutant of SLX4 disrupts the localization of XPF, MUS81, or SLX1 to telomeres. HeLa1.2.11 cells transiently expressing HA-tagged wild-type or L1022A mutant of SLX4 were cotransfected with Myc-tagged XPF, MUS81, or SLX1.
**Figure S4. Assessment of T-SCE in Human Cells via CO-FISH** (A) Western blot analysis of SLX4 knockdown by shRNA. (B) SLX4 knockdown by shRNA was confirmed by IF-telomere-FISH. (C) Western blot analysis of protein expression levels in shSLX4-1 knockdown U2OS cells expressing WT or mutant GFP-SLX4. (D) Schematic representation of the CO-FISH assay. Newly synthesized DNA strands were removed, leaving parental strands to be detected by fluorescent-labeled telomeric C-rich or G-rich probes. A chromosome with more than two telomere signals was considered positive for T-SCE. (E) A representative metaphase spread showing DAPI staining (blue), and leading and lagging strand telomere fluorescence signals (red and green, respectively). Representative normal and T-SCE telomeres are shown on the right. Chromosomes with more than two telomere signals by both probes (arrows) are considered to be positive for T-SCE. (F and G) T-SCE frequency in SLX4 depleted U2OS cells (F) transiently expressing WT or mutant SLX4 (G). Error bars: SD from three independent experiments of each genotype (30 metaphases/genotype/experiment). *P value*s: two-tailed Student's t test. (H) T-SCEs in HeLa1.2.11 cells expressing WT or mutant SLX4. Error bars: SD from three independent experiments of each genotype (30 metaphases/genotype/experiment). *P value*s: two-tailed Student's t test.
**Figure S5. Telomere Length Analysis in ALT (U2OS, GM847) and Telomerase-Positive (HeLa1.2.11) Cells, Related to** Figure 4 (A and B) Assessment of telomere length by Q-FISH in U2OS (A) and GM847 (B) cells expressing scramble, shSLX4-1 or shSLX4-2 RNA. Error Bars: SD. *P value*s: one-way ANOVA. (C) Q-FISH analysis of telomere length in HeLa1.2.11 cells expressing SLX4 fused to WT or endonuclease-dead E82A mutant SLX1. Error Bars: SD. *P value*s: oneway ANOVA. (D) Quantification of the cleavage activity of the oligo-based D loop substrate by SLX1_WT_-SLX4_SBR_ or SLX1_E82A_-SLX4_SBR_, related to Figure 4G. For each reaction, the D loop signal intensity was normalized to the reaction without enzyme (lane 2 or 7 in Figure 4G), which was set at 100%. Error bars represent standard deviations from three independent experiments. (E) A representative blot of the TCA assay, probing for T-circle (TC) (upper panel) and Alu DNA as loading control (lower panel). U2OS cells stably expressing shSLX4-1 RNA were transiently transfected with empty vector (Vector), full-length WT or mutant SLX4. U2OS cells expressing scramble RNA is shown as a control. (F) Q-FISH images comparing telomere signals in cells co-expressing TRF2 and WT or the DSBR mutant of SLX4. U2OS cells were transfected with mCherry WT TRF2 and GFP-WT or the DSBR mutant of SLX4. The GFP- and mCherry-positive cells were sorted and metaphase spreads were analyzed by Q-FISH using Cy3-PNA (CCCTAA)_3_ probe. (G) Quantitation of telomere length in HeLa1.2.11 cells. Cells were co-transfected with mCherry-WT TRF2 and GFP-WT or the DSBR mutant of SLX4. GFP and mCherry positive cells were sorted and metaphase spreads were analyzed by Q-FISH. Error Bars: SD. *P value*s: one-way ANOVA. (H). Proposed model of the SLX4-TRF2 assembled telomere maintenance toolkit. SLX4 is required to assemble a nuclease toolkit via unique interactions between SLX4 and each endonuclease. The HxLxP motif of SLX4 directly binds to the TRFH motif of TRF2 to bridge the SLX4-nuclease complex to telomeres. The thus assembled SLX4-nuclease toolkit regulates telomere HR (T-SCE and t-loop resolution).
