# Supplementary material for: SLX4 Assembles a Telomere Maintenance Toolkit by Bridging Multiple Endonucleases with Telomeres
Source: Cell Rep. Author manuscript; Available in PMC 2015 Feb 19. (PMC4334113; doi:10.1016/j.celrep.2013.08.017)
Supplement: 01 — Figure S1. SLX4 Foci Colocalize with Telomeres, Related to Figure 1 (A) SLX4 forms discrete foci that colocalize with telomeric DNA and TRF2 in ALT cells. IF and IF-telomere FISH were performed using anti-SLX4 and anti-TRF2 antibodies and the PNA (CCCTAA)3 probe. (B) ChIP analysis of SLX4 at telomeric DNA or a control locus Alu in U2OS and HeLa1.2.11 cells. (C) Western blot analysis of endogenous SLX4 expression in human cells. Figure S2. Characterization of the SLX4-TRF2 Interaction, Related to Figure 2 (A) SLX4 constructs used for mapping the interaction with TRF2. AD: activation domain. BD: DNA-binding domain. (B–D) Yeast two-hybrid assay. SLX4 directly interacts with TRF2 but not RAP1 (B); Identification of the specific interaction regions of SLX4 and TRF2 (n = 3; error bars, standard deviations) (C-D). (E and F) ITC measurement of protein-protein interactions. TRF2TRFH and various SLX4 fragments (E); TRF1TRFH and SLX4TBM peptide (St8) (F). Kd: equilibrium dissociation constant; N.D.: not detectable by ITC. (G) Simulated annealing omit map of the SLX4TBM peptide in the SLX4TBM-TRF2TRFH complex. The omit map is contoured at 2.5 s and colored in cyan. The SLX4TBM peptide is shown in magenta stick model and TRF2TRFH in ribbon model. (H and I) ITC measurement of mutant SLX4TBM-TRF2TRFH interactions. SLX4TBM mutants and wild-type TRF2TRFH (H); wild-type SLX4TBM and TRF2TRFH F120A mutant (I). Kd: equilibrium dissociation constant; N.D.: not detectable by ITC. (J) SLX4L1022A or TRF2F120A do not co-localize (IF), even though TRF2F120A mutant localizes to telomeric DNA (IF-telomere FISH). HeLa1.2.11 cells transiently expressing HA-tagged TRF2F120A were co-transfected with either GFP-WT or mutant SLX4 fusion proteins. IF and IF-telomere FISH were performed using anti-HA antibody and the PNA (CCCTAA)3 probe. (K) Endogenous SLX4 colocalizes with wild-type TRF2, but not TRF2F120Amutant. U2OS and HeLa1.2.11 cells were transiently transfected with either HA-tagged wild-type or [file NIHMS526006-supplement-01.pdf]

Table S1 Crystal data collection and refinement statistics

	$TRF2_{TRFH}$-$SLX4_{TBM}$ (4M7C)
**Data collection**	
Space group	$P2_1$
Cell dimensions	
a, b, c (Å)	32.591, 69.183, 118.078
α, β, γ (°)	90, 94.73, 90
Resolution (Å)	50-2.05
R_{merge}	0.068 (0.287)*
I / σI	25.2 (4.7)*
Completeness (%)	96.7 (95.6)*
Redundancy	3.9 (3.7)*
**Refinement**	
Resolution (Å)	44.86-2.05
No. reflections	31,784
R_{work} / R_{free} (%)	21.4/26.2
No. atoms	
$TRF2_{TRFH}$	3,180
$SLX4_{TBM}$	214
Water	271
B-factors (Å^2)	
$TRF2_{TRFH}$	23.39
$SLX4_{TBM}$	30.65
Water	39.17
R.m.s. deviations	
Bond lengths (Å)	0.004
Bond angles (°)	0.866
Ramanchandran plot (%)	
Favored region	98.0
Allowed region	100.0
Outlier region	0.0

* Values in parentheses are for the highest-resolution shell.
